# The reliability and validity of gait speed with different walking pace and distances against general health, physical function, and chronic disease in aged adults

**DOI:** 10.20463/jenb.2016.09.20.3.7

**Published:** 2016-09-30

**Authors:** Hee-jae Kim, Ilhyoek Park, Hyo joo Lee, On Lee

**Affiliations:** 1 Institute of Sport Science, Seoul National University, Seoul Republic of Korea; 2 Physical Activity and Performance Institute, Konkuk University Republic of Korea

**Keywords:** gait speed, Reliability, Validity, Walking test

## Abstract

**[Purpose]:**

Gait speed is an important objective values associated with several health-related outcomes including functional mobility in aging people. However, walking test methodologies and descriptions are not standardized considering specific aims of research. This study examine the reliability and validity of gait speed measured at various distances and paces in elderly Koreans.

**[Methods]:**

Fifty-four female participants ≥70 years of age were recruited from a local retirement community. Gait speed was assessed at 4, 6 and 10 meters, and at usual- and fast-pace walking mode. The short physical performance battery (SPPB) that estimates senior fitness includes three tests of lower-body function. Data concerning for the chronic conditions and self-perceived health of the participants was collected using questionnaires. Concurrent validity of gait speed using the aforementioned test protocols was determined by calculating the Pearson correlation coefficients.

**[Results]:**

Significant positive correlations were evident between skeletal muscle mass and maximal pace walking regardless of distance (r=.301~.308; p<.05), but not with body fat. All gait tests significantly positively correlated with self-rated health (normal pace r=.328~.346, p<.05; maximal pace r=.427~.472, p<.001) and depression (normal pace r=.279~.430, p<.05; maximal pace r=.413~.456, p<.001).

**[Conclusion]:**

Walking test at the normal pace appears suitable for estimating physical function and deterioration due to chronic disease. Walking test at a maximum pace might be useful for estimating subjective general health and skeletal muscle mass.

## INTRODUCTION

Gait speed is an important objective measure of functional mobility, particularly for older adults. The significance of gait speed lies on its relationship to various health outcomes, such as functional decline, discharge location and mortality^1^^-^^3^. Gait speed is also a potentially useful factor to predict future functional decline, rehabilitations and fear of falling^4^^,^ ^5^. Gait speed is frequently used for evaluation of disability in clinical intervention trials and daily settings^6^. Moreover, gait speed can be quickly and easily measured, it is frequently included in research study^7^. Gait speed has been described as a reliable and valid measurement for seniors’ walking performance and is regarded as a pivotal factor associated with the quality of life^8^.

Gait speed has not been assessed in a standardized fashion. Graham et al.^9^ reported that clinical assessments of walking velocity are not conducted uniformly and that common methodologic factors might influence the clinical interpretation of gait performances. Marked variations have been described in gait speed test methodology within both clinical practice and published research. Although most gait speed test methods have excellent interrater and test-retest reliabilities, there is no consensus regarding the optimal measurement protocol including walking distance, instructed pace and start mode^9^.

Gait test distance is marginally related to the mean velocity in the elderly. In general, 4, 6 and 10 meters are used for short-distance walk test for elderly adults. This discordance in walking length has led to confusion concerning the optimal measuring method for gait speed. Differences in gait speed between usual- and fast-paced tests within the same participant group have been described. Graham et al.^9^ reported that the intended pace significantly affects mean gait velocity in elderly individuals. Additionally, the dynamic start mode effectively eliminates the acceleration phase from the timed performance. In prior research, the dynamic protocol showed greater mean velocity compared with static-start conditions, although the difference was not significant.

In the elderly, few studies have examined how subtle differences in test walking distance and walking pace affect gait speed reliability and validity. It is uncertain whether a longer walking distance produces a more accurate determination of gait speed than a shorter distance. Presently, we examine the reliability and validity of the gait speed in elderly Koreans. Intraclass correlation coefficient (ICCs) was evaluated to examine test-retest reliability of variant walking distances (4, 6 and 10 meters) and walking pace (usual- or fast-pace). We also evaluated the validity of gait speed using protocols featuring varied physical function, body composition and presence of depression.

## METHODS

### Design and participants

A cross-sectional study design was used to compare different gait speed measurements including walking distance and walking pace. Sixty-five female participants were recruited from a local retirement community. Inclusion criteria included age ≥70 years, ability to reliably follow two-step instructions and the ability to walk 100 meters with or without an assistive device. Exclusion criteria included severe visual impairment and/or severe arthritis or orthopedic problems that limited ambulation. All participants gave written informed consent. The Seoul National University Institutional Review Board approved this study (SNUIRB 1210/001-003).

### Body composition and blood pressure

Anthropometric parameters were screened by the same examiner. Height was evaluated using an extensometer. Body weight, body mass index (BMI), fat mass, percent body fat, fat free mass and skeletal muscle mass were measured by bioimpendence analysis using an Inbody 370 (Biospace, Korea). Blood pressure was measured at resting condition using a Biospace- BPBIO320.

### Walking procedure and measurement

We assessed gait speed over 4, 6 and 10 meters, and usual- and fast-pace walking mode. The test was repeated twice with the mean of the two trials used for scoring purposes. Participants were instructed to walk from a standing start at a pace that was normal and comfortable for them or to walk as fast as they could until they reached the end of the marked path. A trained tester walked behind the participant and stopped timing when the participant’s foot contacted the floor at the end of the walking course. Participants were provided rest breaks as needed throughout the testing session.

### Short physical performance battery (SPPB) measurement

The SPPB includes three components of lower-body function: a hierarchical test of standing balance, a 4-meter walk and five repetitive chair stands^10^. Each SPPB component test is scored from 0 to 4 with a score of 0 representing inability to perform the test and a score of 4 representing the highest category of performance, with scoring cut-points derived from a large representative population of older persons^11^. For the balance tasks, the participants were asked to stand with their feet side-by-side, followed by the semi-tandem (heel of one foot alongside the big toe of the other foot) and tandem (heel of one foot directly in front of and touching the other foot) positions for 10 seconds each. For gait speed, a 4-meter walk at the participant’s usual pace was timed. For those who did not have 4 meters of space available in their homes, a 3-meter course was used and scoring was modified as indicated in the instructions. The test was repeated twice with the faster of the two walks used. For the ability to rise from a chair, participants were asked to stand up and sit down five times as quickly as possible with arms folded across their chests. This was done only after participants first demonstrated the ability to rise once without using their arms. Further details on the administration of these tests have been published^10^. A summary performance score was obtained by adding the scores of each individual SPPB component test (range 0-12), with higher scores indicating better lower-body function^11^.

### Questionnaires

The data for the chronic conditions and health self-perception of participants was collected using questionnaires. Perception of general health was self-reported with a score ranging from 1 to 10 points. Chronic conditions were assessed by self-reporting as having been diagnosed by a medical doctor for hypertension, heart diseases, diabetes, cancer, chronic respiratory disease, arthritis or depression. Chronic diseases including cancer and chronic respiratory disease were excluded because of the extremely low number of cases.

### Statistical analysis

Statistical analysis were processed using SPSS 18.0 (Statistics Package for Social Science, Ver. 18.0 for Windows; SPSS Inc., Chicago IL, USA). ICCs were used to estimate test–retest reliability. We determined the concurrent validity of the gait speed with variant test protocols by calculating the Pearson correlation coefficients between body composition, self-rated health, depression and physical function assessed by combinations of the SBBP components.

## RESULTS

Subject characteristics are shown in Table 1. All subjects were females ≥70 years of age and able to walk independently. Table 2 shows the estimates of test–retest reliability for gait tests with different distance and pace. ICC for the 4-, 6- and 8-meter gait test was r= .715, .837; r=.861, .905; and r=.902, .933, respectively. Reliability increased with gait distance and speed. There was no significant difference in the gait tests each participant walked between the first and second tests.

**Table 1:** Characteristics of participants

N=65	Mean ± S.E.M
Age (years)	77.70 ± 4.8
Height (cm)	151.09 ± 4.5
Body weight (kg)	55.90 ± 7.0
Skeletal muscle mass (kg)	20.04 ± 5.0
Body fat percentage (%)	35.90 ± 5.6
Systolic blood pressure (mmHg)	138.66 ± 73.0
Diastolic blood pressure (mmHg)	70.98 ± 8.6

**Table 2:** Reliability of walking test (ICC)

	Normal pace			Maximal pace		
	4 M	6 M	10 M	4 M	6 M	10 M
ICC (95% CI)	.715^**^	.861^**^	.902^**^	.837^**^	.905^**^	.933^*^

Table 3 presents the Pearson’s correlation coefficients used to assess the validity of gait tests with the three distances and two walking paces according to body composition, self-rated health and depression. Significant positive correlations were evident between skeletal muscle mass and maximal pace walking regardless of distance (r=.301~.308; p<.05), but not for body fat. All gait tests were significantly positively correlated with self-rated health (normal pace r=.328~.346, p<.05; maximal pace r=.427~.472, p<.001) and depression (normal pace r=.279~.430, p<.05; maximal pace r=.413~.456, p<.001). The correlation score increased with gait distance and maximum speed walking produced a higher score than normal speed in both variables.

**Table 3:** Validity of walking speed with different measuring protocols against health-related variables

		Normal pace			Maximal pace		
		4 M	6 M	10 M	4 M	6 M	10 M
SPPB (Gait + Balance + Chair)	R (Pearson)	.735	.677	.740	.563	.592	.601
	p	<.001	<.001	<.001	<.001	<.001	<.001
Balance + Chair	R (Pearson)	.554	.506	.568	.531	.495	.536
	p	<.001	<.001	<.001	<.001	<.001	<.001
Self-rated health	R (Pearson)	.328	.346	.346	.427	.452	.472
	p	.008	.002	.005	<.001	<.001	<.001
Body Fat (%)	R (Pearson)	.031	.063	.034	.071	.053	.022
	p	.804	.616	.789	.573	.674	.864
Skeletal muscle mass (%)	R (Pearson)	.074	.055	.097	.303^*^	.308^*^	.301^*^
	p	.556	.662	.442	.014	.013	.015
Depression score	.281	0.279	0.322	0.430	0.413	0.446	0.456
	<0.05	<0.05	<0.01	<0.001	<0.001	<0.001	<0.001

Table 3 summarizes the correlations between various gait tests and combination of SBBP components. All gait tests were significantly positively correlated with a combination of SPPB components. There were moderate to high correlations between the various gait tests and SPPB total score: 4-meter walk (r=.735, .563), 6-meter walk (r=.677, .592) and 10-meter walk (r=.740, .601). There were moderate correlations between various gait tests and combination of SPPB components (sum of balance and chair score): 4-meter walk (r=.554, .531), 6-meter walk (r=.506, .496) and 10-meter walk (r=.568, .536). In contrast with results from Table 3, normal speed walking produced a higher score than maximal speed walking. Four-meter walk at a normal pace produced the second highest score among all values.

## DISCUSSION

In the present study, we examined the reliability and validity of the gait speed with various walking pace and distance in aged group. The validity of gait speed with normal pace was higher than that with maximal pace against physical function. Relatively higher values of validity were found at maximal walking pace against perception of general health and skeletal muscle mass. In addition, although there was no statistical difference between gait speed and chronic diseases, there was a tendency that gait speed at normal pace showed a higher validity than that at maximal pace. Taken together, the results suggest that an appropriate protocol of walking test could be differently applied by specific research purpose.

Both floor and ceiling effects for measures of gate speed have been reported in patient groups suggesting that a short-distance walking test has a narrow range of applications^11^^-^^13^. Although there are considerable variations in testing methods, measurement of the gate speed is valid and sensitive outcome measurement in a broad range of people.^13^^-^^16^. Therefore, the study to assess gate-test methodology including walking distance and pace was needed in aged population.

The ICC values were similar to those reported in studies evaluating reliability of gait test (0.88 to 0.97) in aging populations^16^^-^^19^. As shown in Table 2, higher ICC values were observed at the longest walking distance of 10 meters compared to 4 and 6 meters. In addition, ICC values of gait test at maximal speed were higher than that at the normal pace (Table 2). Although the walking test at a maximal pace over a longer distance has better reliability in elderly individuals, test distance and pace have to be considered according to the purpose of the measurements and the clinical health conditions of participants, rather than by the criterion of a high level of reliability.

Concurrent validity of walking test with various walking distances and paces was examined against physical function, measures of general health, body composition and chronic diseases in the elderly participants. Physical function evaluated as SPPB scores positively correlated with walking speed without regard to walking distance or pace (Table 3). Since SPPB scores including 4-meter walking speed, the high correlation between SPPB score and walking speed was inevitable. Therefore, we additionally estimated the relationship between walking speed and SPPB scores excluding the 4-meter walking score (scores of balance test and chair stand). High correlation between SPPB scores excluding 4-meter walking test score and walking speed was found, whereas there was no difference among all of the test groups. These results suggest that walking test regardless of test distance or walking pace is positively associated with physical function in aged adults. Furthermore, it might be possible to use gait speed to estimate physical function in elderly individuals when test conditions including space or time are limited in clinical setting.

Walking speed at a maximal pace was associated with better subjective evaluation for the general heath (self-rated health). A similar tendency was found in the relationship between skeletal muscle mass and walking speed with maximal pace, but no association was apparent between body fat and maximal walking speed. In healthy aged individuals, decreased muscle mass and muscle strength in the lower extremities affects walking speed and can affect daily physical activities^19^^,^ ^20^. Therefore, measurement of walking speed at maximal pace could be the one of the most efficient variable for predicting the health conditions following decrease of muscle functions in aging population.

Differences in walking speed between normal pace and maximal pace with in the same participants have been described^20^^-^^23^. It is important to note that in prior studies normal (usual and/or comfortable) pace was used approximately twice as often as maximal pace^8^^,^ ^9^, and that normal pace was considered the more common normative value than maximal pace standards^17^^,^ ^18^. Both normal and maximal pace walking measurement are important and that the difference between normal and maximal walking velocities (i.e., the ability to voluntarily increase walking velocity) may be the best indicator of community-based ambulation ability in aged adults^9^^,^ ^20^. Presently, several values including subjective estimate of general health, depression statues and skeletal muscle mass showed higher correlation with maximal pace than normal pace. Considering the results of relatively lower association with less than 10-meter walking distance, the appropriate protocol of walking pace needs more deliberation than walking distance in the specific research purpose.

This study is one of the few to investigate the suitable methods of gait test for the valid indicator of physical function and general health in aging population. However, several limitations of the present study are noted. These are the relatively small sample size, disproportion sex representation and lack of medical examination. Future studies evaluating the validity of elderly walking test against health including chronic disorders will be needed and will require a large number of participants.

In conclusion, our data reveals a high level of validity was found following the walk speed test with different distance or pace against various health related factors in aged adults. Based on our results and previous review articles, walking test at the normal pace might be adopted for estimating physical function and chronic disease, whereas walking test at the maximal pace might be adopted for estimating subjective general health and skeletal muscle mass. Additional studies are needed to determine if differences in walk testing methods will yield predictable and meaningful differences in the distribution of performance scores.
